# Design and modeling of high-performance mid-wave infrared InAsSb-based nBn photodetector using barrier band engineering approaches

**DOI:** 10.1007/s12200-023-00060-9

**Published:** 2023-04-06

**Authors:** Maryam Shaveisi, Peiman Aliparast

**Affiliations:** Aerospace Research Institute (Ministry of Science, Research and Technology), Tehran, 1465774111 Iran

**Keywords:** Mid-wave infrared detectors, III-V compound semiconductors, Grading material systems, nBn architecture

## Abstract

We report a new nBn photodetector (nBn-PD) design based on the InAlSb/AlSb/InAlSb/InAsSb material systems for mid-wavelength infrared (MWIR) applications. In this structure, delta-doped compositionally graded barrier (δ-DCGB) layers are suggested, the advantage of which is creation of a near zero valence band offset in nBn photodetectors. The design of the δ-DCGB nBn-PD device includes a 3 µm absorber layer (n-InAs_0.81_Sb_0.19_), a unipolar barrier layer (AlSb), and 0.2 μm contact layer (n-InAs_0.81_Sb_0.19_) as well as a 0.116 µm linear grading region (InAlSb) from the contact to the barrier layer and also from the barrier to the absorber layer. The analysis includes various dark current contributions, such as the Shockley–Read–Hall (SRH), trap-assisted tunneling (TAT), Auger, and Radiative recombination mechanisms, to acquire more precise results. Consequently, we show that the method used in the nBn device design leads to diffusion-limited dark current so that the dark current density is 2.596 × 10^−8^ A/cm^2^ at 150 K and a bias voltage of − 0.2 V. The proposed nBn detector exhibits a 50% cutoff wavelength of more than 5 µm, the peak current responsivity is 1.6 A/W at a wavelength of 4.5 µm and a − 0.2 V bias with 0.05 W/cm^2^ backside illumination without anti-reflective coating. The maximum quantum efficiency at 4.5 µm is about 48.6%, and peak specific detectivity (D*) is of 3.37 × 10^10^ cm⋅Hz^1/2^/W. Next, to solve the reflection concern in this nBn devices, we use a BaF_2_ anti-reflection coating layer due to its high transmittance in the MWIR window. It leads to an increase of almost 100% in the optical response metrics, such as the current responsivity, quantum efficiency, and detectivity, compared to the optical response without an anti-reflection coating layer.

**Graphical Abstract:**

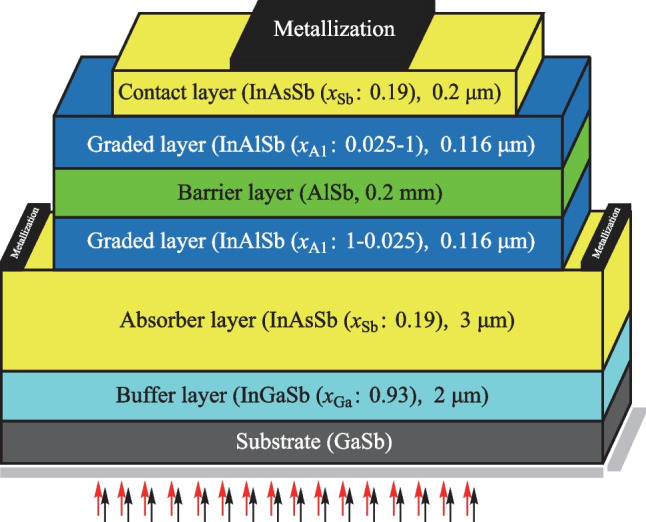

## Introduction

The InAsSb infrared detectors show the highest performances among all infrared detectors due to their excellent optical and electrical properties, which is the reason why the use of the InAsSb material in the semiconductor industry has a long history [1]. The InAsSb material has the lowest band gap energy among group III-V semiconductors. It can be used as an active region material for designing mid-wavelength infrared (MWIR) and long-wavelength infrared (LWIR) optical detectors [2, 3]. However, the limitations of conventional PN or PIN photodiodes operating under high operating temperature conditions indicate that the dark current is mainly generated by Shockley–Read–Hall (SRH) generation-recombination (GR), Auger recombination, band-to-band tunneling (BTBT) and trap-assisted tunneling (TAT) processes [4]. Also, with avalanche photodiodes (APDs) using the impact ionization process, it is possible to achieve higher sensitivity than with conventional photodetectors. However, these detectors also show a high dark current due to the carrier recombination effect [5]. Previous studies suggest that a wide band gap material used as a barrier between the contact layer and the absorber layer can expand the operating temperature, reducing the dark current caused by the majority of carriers [6]. Among the reported barrier detectors, the nBn photodetectors have received the most attention in commercialization [7–13]. In 2006, Maimon and Wicks introduced nBn detectors and published one of the most influential works [14]. This work states that the performance of nBn photodetector is controlled by a barrier layer. The barrier layer material is a semiconductor with a large band gap energy, located between the two contacts and the narrow band gap absorber layer. Majority carriers are blocked, but there is no barrier for minority carriers to pass. These unipolar barriers are used to suppress the two main dark current mechanisms, GR dark current and surface leakage current [15]. First infrared nBn detectors were built by the growth of the InAs absorber layer on the InAs substrate and also the growth of the InAs_0.91_Sb_0.09_ alloy on the lattice-matched GaSb substrate [14]. These devices have cutoff wavelengths of 3.2 and 4 µm, respectively. Like InSb infrared photodetectors, they do not cover the whole mid-infrared wavelength range (3–5 µm) but can operate at higher temperatures than InSb detectors [16]. Ting et al. have modeled the standard InAsSb-based nBn photodetector described by Maimon and Wicks. They have investigated the effects of doping in the contact and absorber layers and minority carrier lifetimes which affect the dark current of the proposed nBn structure. Their study showed that the dark current can be reduced by proper design, and the maximum quantum efficiency can be obtained [17]. For achieving optimal performance in InAsSb-based nBn structures, the barrier layer must be designed to create the maximum conduction band offset (CBO) and the minimum valence band offset (VBO). However, finding a material with a large band gap, a large CBO, and a slight VBO is complicated. Previous studies have proposed several band engineering methods to compensate for the valence band discontinuity problem in nBn structures. Among them, the most common ones are the simultaneous grading doping concentration and material composition, and barrier layer methods based on semiconductor superlattice [12, 13, 18, 19]. Recently, Deng et al. have reported a new InAsSb-based nBn detector with AlAs_0.08_Sb_0.92_/AlSb compound barrier layer [20]. By designing a 100 nm thick AlAs_0.08_Sb_0.92_/AlSb compound barrier layer, they were able to reduce the VBO in nBn detectors. The simulation results have confirmed that the dark current caused by GR processes, tunneling, and surface leakage currents were effectively suppressed. In addition, the operating temperature was raised to about 205 K. In another study, Deng et al. have suggested an MWIR photodetector based on pCBn architecture [21]. They have utilized short-period AlAs_0.08_Sb_0.92_/AlSb (4/1.5 mono-layers (MLs)) superlattices with a thickness of 150 nm as the barrier. This compound barrier layer has created a lower VBO and higher CBO than is achieved by the AlAs_0.08_Sb_0.92_ barrier. The simulation results have shown that the device has good high-temperature performance, and the main mechanisms of dark current can be relatively suppressed.

In this study, we introduce a new infrared photodetector device called a delta-doped compositionally graded barrier nBn photodetector (δ-DCGB nBn-PD) to minimize the VBO in InAs_0.81_Sb_0.19_ based nBn structures while maximizing the CBO. The AlSb layer, a typical barrier, has a VBO for the InAsSb layer with a Sb composition above 15% [22]. This method, which is developed based on δ-doped layers in the vicinity of the barrier layer, is used together with a compositionally graded barrier layer. This method has been successfully applied to InGaAs and HgCdTe unipolar detectors [23, 24], but so far it has not been implemented for InAsSb nBn detectors. The proposed method used to reduce the VBO in this structure is more convenient than previously presented methods, and it can be fabricated by using the molecular beam epitaxy (MBE) technique. A benefit of this approach is that it does not require simultaneous grading of n- and p-type doping profiles. Another critical issue is that in focal plane arrays (FPAs), which is usually irradiated from the back, infrared light is absorbed through the substrate on which the detector array is grown. Besides, conventional detectors have significant reflection losses at the air/semiconductor interface, so an anti-reflection coating must be included to reduce these losses. Unfortunately, it is complicated to engineer an anti-reflection coating that works effectively over the entire spectral range with very low reflection intensity. In this design, we propose the use of BaF_2_. It gives no external reflection and has significant transmission ability in the MWIR range. Based on the simulation results, it can be said that this approach significantly increases the responsivity and quantum efficiency of the nBn detector.

## Simulation approach and IR-detector design

**Fig. 1:**
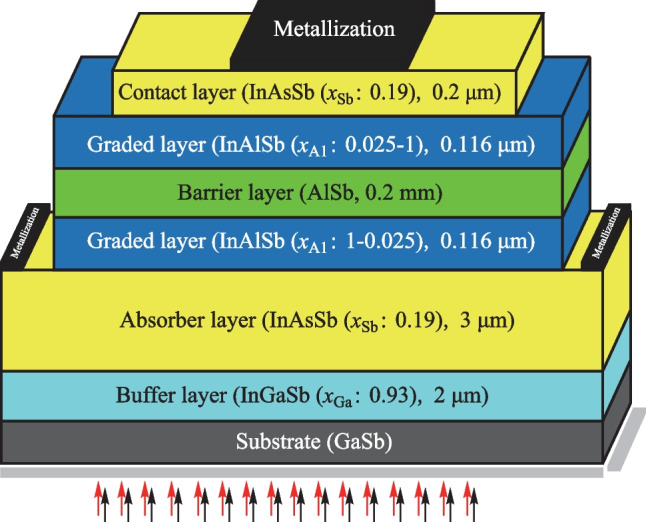
Schematic of the proposed InAsSb nBn photodetector structure

As seen in Fig. 1, the proposed nBn photodetector is designed based on the compositionally graded barrier (CGB) method that employs the InAlSb material system. The main layers of the proposed device, which has a 30 µm × 30 µm area, can be grown on the GaSb:Te(100) substrate. The epilayers of the structure start with a buffer layer of 2 µm InGaSb, with n-type doping of 1 × 10^15^ cm^−3^. It is used to provide better lattice matching between the layers. Then an n-type InAs_0.81_Sb_0.19_ absorber layer with a thickness of 3 µm is placed, with a doping level of 3 × 10^15^ cm^−3^. On top of the absorber layer, a 0.116 µm grading layer of InAlSb should be grown. Then, the n-type AlSb barrier layer with a thickness of 0.2 µm and doping of 1 × 10^16^ cm^−3^ is positioned. The thickness of the barrier layer is sufficient to prevent electron tunneling between the contact layer and the absorber layer. Similarly, another InAlSb layer with a thickness of 0.116 µm is placed as the top-grading layer. In the end, the InAs_0.81_Sb_0.19_ contact layer is also designed with a thickness of 0.1 µm and doping density of 1 × 10^16^ cm^−3^.

The design parameters of the device discussed in this paper are listed in Table 1. In the simulation, the use of a detailed material model is required, and some important parameters are collected from literatures [25–28]. The structures of the proposed detectors are modeled using the Atlas module in the Silvaco device simulator. This tool uses a set of material properties to characterize the III-V semiconductors, such as energy band gap, electron affinity, dielectric constant, electron and hole mobility, conduction and valance band states densities, and others. In the simulation, we set the parameter values based on the equations and data reported by the Ioffe physical-technical institute (see the website of ioffe.ru). We use the Blaze tool to plot the band structure, which is interfaced with the Atlas module. In addition, the optical response of the device is analyzed using the Luminous tool in the Atlas simulator. Luminous is an advanced device simulator capable of modeling light absorption and generation in III-V semiconductor devices. The absorption coefficient α is dependent on wavelength (or photon energy *E*), temperature *T* and mole fraction *x* of absorber layer, and can be computed for the InAsSb-based detector using Eqs. (1) and (2). Further considerations about the optical properties can be found in the literature reported by D’souza et al. [29]. If the photon energy is less than the energy band gap:

1 $$\begin{gathered} \alpha (E,x,T) = 948.23 \times {\text{e}}^{{170(E - E_{0} )}} \,, \hfill \\ E_{0} = E_{{\text{g}}} + 0.001\,.\,\,\,\,\,\,\,\,\,\,\,\,\,\,\,\,\,\,\,\,\,\,\,\,\,\,\,\,\,\,\, \hfill \\ \end{gathered}$$

**Table 1:** Design parameters of the proposed nBn photodetector used in the simulation

Layer	nBn structure
Contact	
Material	InAsSb (*x*_Sb_: 0.19)
Doping/cm^−3^	n: 3 × 10^15^
Thickness/µm	0.2
Grading	
Material	InAlSb (*x*_Al_: 0.025–1)
Doping/cm^−3^	n: 1 × 10^16^
Thickness/µm	0.116
δ-doping	
Material	InAlSb (*x*_Al_: 0.025,1)
Doping/cm^−3^	n: 7 × 10^15^, p: 1 × 10^17^
Thickness/nm	2
Barrier	
Material	AlSb
Doping/cm^−3^	Undoped
Thickness/µm	0.2
Absorber	
Material	InAsSb (*x*_Sb_: 0.19)
Doping/cm^−3^	n: 3 × 10^15^
Thickness/µm	3
Buffer	
Material	InGaSb (*x*_Ga_: 0.93)
Doping/cm^−3^	n: 3 × 10^17^
Thickness/µm	2
Substrate	
Material	GaSb
Doping/cm^−3^	n: 5 × 10^17^
Thickness/µm	10

While if the photon energy is greater than the energy band gap:

2 $$\begin{gathered} \alpha (E,x,T) = \frac{{\left[ {K(E - E_{{\text{g}}} - c)\sqrt {(E - E_{{\text{g}}} - c)^{2} - c^{2} } } \right]}}{E}\, + 800\,, \hfill \\ K = 10000 + 20000E_{{\text{g}}} \,, \hfill \\ c = 0.1 + 0.5E_{{\text{g}}} \,.\, \hfill \\ \end{gathered}$$

The following equation is used to model the energy band gap *E*_g_ of the InAsSb compound, which is dependent on temperature (*T*, measured in K) and mole fraction of Sb (*x*_Sb_) [30]:

3 $$E_{{\text{g}}} (x_{{{\text{Sb}}}} ,T) = 0.411 - \frac{{3.4 \times 10^{ - 4} T^{2} }}{210 + T} - 0.876x_{{{\text{Sb}}}} + 0.70x_{{{\text{Sb}}}}^{{2}} + 3.4 \times 10^{ - 4} x_{{{\text{Sb}}}} T(1 - x_{{{\text{Sb}}}} )\,.\,$$

The energy band gap of the InAlSb compound is modeled based on Eq. (4) [31]:

4 $$E_\text{g}(x_\text{Al},T)=0.235-\frac{3.2\times10^{-4}T^2}{T+170}+1.721x_\text{Al}\,...\,-\left(\frac{4.2}{T+140}-\frac{3.2}{T+170}\right)10^{-4}T^2x_\text{Al}\,+0.43x_\text{Al}^2\,.\,$$

Solving Poisson’s equation and using the drift–diffusion model, which includes electrical and optical characteristics, makes it possible to model the device accurately in Silvaco software. For more accurate modeling of nBn detector performance, models for SRH, Auger, radiative, and tunneling recombination are also considered. In our simulations, the recombination parameters, such as the SRH carrier lifetime (*τ*_n_ and *τ*_p_), the Auger recombination coefficient, the radiative recombination coefficient, and the activation parameters of donor traps, are modeled using the data given in Table 2 [32, 33].

**Table 2:** Recombination and defect parameters of the nBn photodetector used in the simulation

Parameter	Symbol	Value		Unit
		Absorber/contact	Compound barrier	
Electron lifetime	*τ*_n_	0.5	1	µs
Hole lifetime	*τ*_p_	1	1	µs
Trap energy level	*E*_Trap_	0.25*E*_g_	0.5*E*_g_	eV
Trap concentration	*N*_Trap_	1 × 10^9^	1 × 10^5^	cm^−3^
SRH capture cross-section	*σ*_n_/*σ*_p_	5 × 10^−15^	5 × 10^−15^	cm^2^
Electron Auger recombination coefficient	*C*_n_	5 × 10^−27^	0	cm^6^·s^–^^1^
Hole Auger recombination coefficient	*C*_p_	1 × 10^−27^	0	cm^6^·s^–1^
Radiative recombination coefficient	*B*	8.9 × 10^−11^	0	cm^3^·s^–1^

**Fig. 2:**
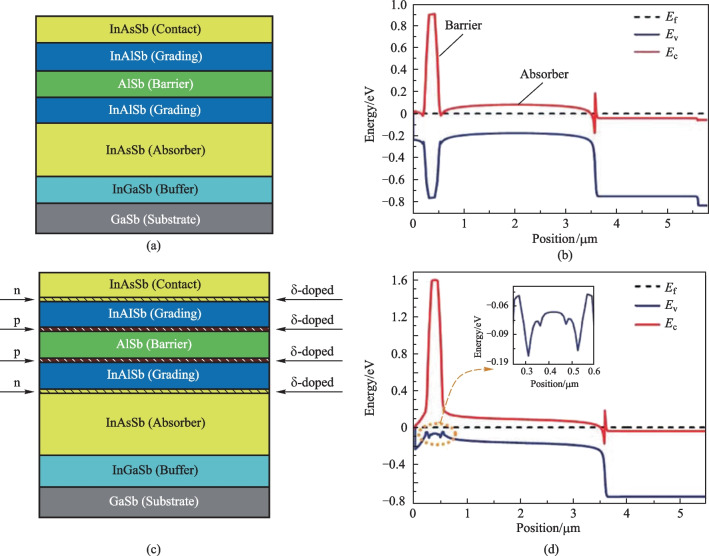
InAsSb-Based nBn IR-Photodetectors; **a** Device structure and **b** energy band diagram with compositionally graded barrier (CGB) layer. **c** Device structure and **d** energy band diagram with δ-doped compositionally graded barrier (δ-DCGB) layer. The position in the vertical direction is calculated by taking the upper surface of the device as the reference

Also, Refs. [27, 30, 32] provide more information about proposed recombination models, especially the tunneling models. Here, the compositionally graded InAlSb barrier layer is obtained by gradually changing the mole fraction of Al in the InAlSb to grade from a narrow band gap material to a wideband gap material while maintaining the lattice constant. As seen in Fig. 2a, b the band structure without band engineering is contrary to that of the ideal design. In addition to the conduction band barrier, there is also a barrier for minority carriers in the valence band. A band engineering method is presented in this study to remove the barrier of minority carriers. In this method, n and p δ-doped nanolayers are placed on both sides of the graded barrier layers, with p-type δ-doped nanolayers at the interface with wide band gap material, and p-type δ-doped nanolayers are placed at the interface with narrow band gap material. Therefore, the electric field caused by the n-type and p-type δ-doping overcomes the quasi-electric field, and the valence band offset is minimized. As a result, an ideal band structure of the nBn detector is obtained, as shown in Fig. 2c, d. This method is beneficial for creating the required barrier layers without the problem of lattice mismatch.

## Result and discussion

**Fig. 3:**
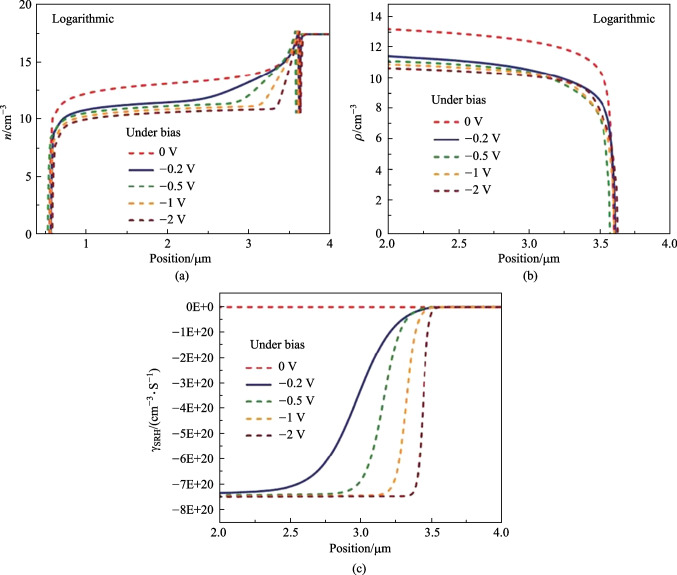
Plots of the results in the absorber layer of the δ-DCGB nBn-PD device as function of the position at various applied bias voltages. **a** Electron density. **b** Hole density. **c** SRH recombination rates (negative value means generation)

**Fig. 4:**
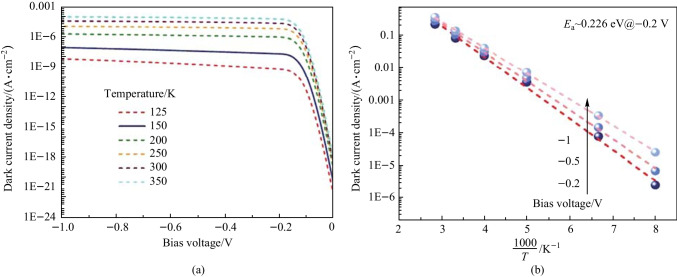
Dark current of δ-DCGB nBn-PD device. **a** Dark current density vs. bias voltage. **b** Arrhenius plot of the dark current density under various applied bias voltages at temperatures from 125 to 350 K

We model the InAsSb nBn detector with the δ-doped compositionally graded barrier layer, which includes contact layers, an upper-graded layer, a barrier layer, a lower-graded layer, an absorber layer, and a buffer layer. The absorber layer has a mole fraction *x*_Sb_ = 0.19 and can be grown on the InGaSb buffer layer at 150 K. This detector works based on the minority carriers, and the transfer of holes from the absorber layer to the contact layer mainly causes the dark current. As we can see from Fig. 3a, the electron density in the absorber-barrier interface region (the grading layer is considered as a part of the barrier) is depleted under significant reverse biases. The electron density decreases, leading to an increase in the SRH rate with an increasing reverse bias voltage (see Fig. 3c). Figure 3b shows that when using a large reverse bias voltage, the density of minority carriers decreases. Figure 4a depicts the current–voltage characteristic curves of the nBn structure simulated at different temperatures in the range of 125–350 K. Under − 0.2 V bias, the nBn photodetector shows a dark current of 2.596 × 10^−8^ A/cm^2^ at 150 K. While at the *T* = 300 K, the dark current increases to 2.786 × 10^−5^ A/cm^2^. Figure 4b shows the Arrhenius plot of dark current versus temperature reversal (1000/*T*) at a bias voltage of − 0.2 V. In the temperature range *T* = 125–350 K, we can see that the temperature dependency of the dark current can be nicely modeled by the equation for diffusion dominated dark current $J_{{{\text{diff}}}} \approx T^{3} \exp ( - E_{{\text{a}}} /(k_{{\text{B}}} T))$ and the activation energy *E*_a_ = 0.226 eV, which is obtained from fitting the dark current data and is almost equal to the bandgap energy of the absorber layer. The dominance of diffusion mechanism confirms the limitation due to dark current in device performance. Increase of the reverse bias voltage is associated with a decrease in the activation energy, indicating greater participation of the GR process and tunneling processes in the dark current. In the Arrhenius plot reported in Ref. [34], the authors have reported that at temperatures higher than 170 K, the dark current due to diffusion is dominant. In contrast, at temperatures lower than 170 K, the GR current is dominant, whereby the transition temperature *T*_0_ as the transition point from GR to diffusion mechanisms is defined. It is worth mentioning that the GR current can be suppressed by the optimal design of the graded barrier of the nBn detector and the tunneling current can be prevented by choosing a thick enough barrier, and the thermionic emission current can be also suppressed by the graded design of the barrier layer.

**Fig. 5:**
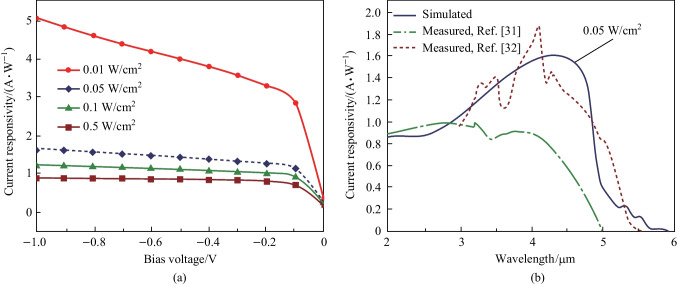
Current responsivity of the δ-DCGB nBn-PD device at 150 K. **a** Responsivity versus applied bias voltages for different incident light intensities. **b** Simulated spectral response compared to the measured values

The optical characterization results of the δ-DCGB nBn-PD device are given in Fig. 5. As can be seen, the 50% cutoff wavelength is greater than 5 µm at 150 K, which covers the wavelength range expected from the MWIR nBn detector design. In Fig. 5a, we have drawn the change of current responsivity with applied bias voltage, when the device is under various intensities of back-illumination radiations. The maximum current responsivity for a bias voltage of − 0.2 V under irradiation of 0.05 W/cm^2^ is 1.6 A/W and occurs at a wavelength of 4.5 µm when the thickness of the absorber layer is 3 µm. In Fig. 5b, the current responsivity of the proposed detector in terms of wavelength is compared with the responses reported for structures reported in Refs. [35] and [36], showing the acceptable performance of the proposed photodetector.

One of the most significant criteria for evaluating the optical response of the nBn detector is the quantum efficiency (QE), which is a function of the incident radiation wavelength *λ*; the current responsivity *R*_i_ can be estimated from the following equation [37]:

5 $$\eta (\lambda ) = 1.24\frac{{R_{{\text{i}}} }}{\lambda }.$$

**Fig. 6:**
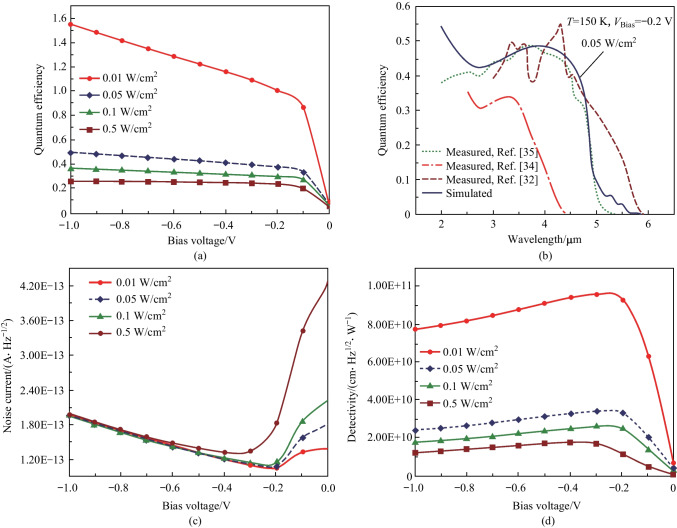
Performance of the δ-DCGB nBn-PD device at 150K. **a** QE versus applied bias voltages with different intensities of infrared radiations. **b** QE versus wavelength compared to the measured values. **c** Noise current. **d** Specific detectivity versus applied bias voltages with different intensities of infrared radiations

In Fig. 6a, the dependence of the quantum efficiency on the bias voltage is shown for different intensities of infrared radiations. The quantum efficiency is approximately 50% at a bias voltage of − 0.2 V, under back illumination with wavelength of 4 µm and radiation power of 0.05 W/cm^2^. In Fig. 6b, we observe that the maximum quantum efficiency achieved at the wavelength of 4.5 µm is approximately 48.6% and the related curve is compared with those for Refs. [36, 38, 39]. The performance of nBn detectors is mainly limited by the shot noise and thermal noise in the dark current (Fig. 6c). This total noise current is the sum of the contributions from shot noise and Johnson thermal noise, and is expressed by the following equation [37]:

6 $$i_{n} (V) = \sqrt {4k_{{\text{B}}} T/RA + 2qJ_{{{\text{Dark}}}} } \,.\,\,$$

In the above noise equation, *R* is the dynamic resistance, *T* is the temperature,* A* is the detector area, and *k*_B_ is Boltzmann’s constant. Correspondingly, specific detectivity (D*) is calculated as an index to evaluate the signal-to-noise ratio of the detector according to the following equation [37]:

7 $$D^{ * } = \frac{{R_{i} }}{{i_{n} (V)}}\sqrt A \,\,.\,\,$$

The simulation results show that for the detector structure designed with absorber doping density of 3 × 10^15^ cm^−3^ and a thickness of 3 µm, the noise current has the lowest value at the bias voltage of − 0.2 V and is about 1.18 × 10^−13^ A⋅Hz^−1/2^ (Fig. 6c).

Figure 6d shows specific detectivity, D*, corresponding to the calculated noise current shown in Fig. 6c, revealing that at a bias voltage of − 0.2 V and intensity of 0.05 W/cm^2^, D* has a peak of 3.37 × 10^10^ cm⋅Hz^1/2^/W. Table 3 presents the performance of the proposed photodetector and other recently reported optical detectors, showings that the proposed photodetector has comparable performance to the state-of-the-art commercial photodetectors.

**Table 3:** Summary of the performance of recently presented MWIR photodetectors

Parameters	Bulk InAsSb nBn [7]	InAs p-i-n photovoltaic [40]	InAs/InAs_0.6_Sb_0.4_ type-II superlattice (T2SL) nBn [41]	InAsSb p-i-n photovoltaic [42]	δ-DCGB InAsSb nBn (This work)
Operating temperature/K	250	300	200	300	150
Cutoff wavelength /µm	4.5	3.5	5.3	< 5	< 5
An applied bias voltage/V	− 0.2	Unbiased	0.05	− 0.5	− 0.2
Specific detectivity /(cm⋅Hz^1/2^⋅W^–^^1^)	5 × 10^9^	6.1 × 10^8^	~ 10^10^	8.9 × 10^8^	3.37 × 10^10^

The main problem in the design of optical devices is the reflection of a large part of the incident radiation on the surface of the device, so providing a solution to reduce unwanted reflections in optical systems should not be neglected. One of the solutions that can solve this problem, increasing the transmission coefficient and reducing the reflection coefficient, is the use of anti-reflective materials as a coating layer on the outer surface. The wavelength, cost, and required performance should be assessed when choosing an anti-reflection coating material for specific applications. For the MWIR range, Silicon (Si) and Germanium (Ge) materials are currently used as anti-reflection materials with high refractive indices and temperature coefficient of refractive indices (d*n*/d*T*).

**Fig. 7:**
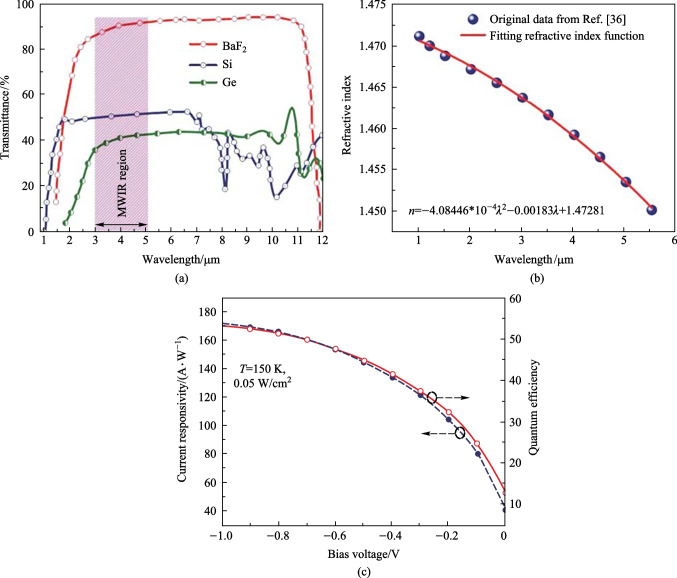
δ-DCGB nBn-PD device with BaF_2_ as the anti-reflection coating material at 150 K. **a** Transmittance of BaF_2_ and current infrared anti-reflection coating materials. **b** Polynomial fitting of the refractive index of BaF_2_. **c** Current responsivity and quantum efficiency versus the applied bias voltage.

As shown in Fig. 7a, Si and Ge have a relatively lower transmittance in the MWIR range. For this reason, we used BaF_2_ as an alternative MWIR anti-reflection coating material. In the simulation, we calculated the refractive index from a polynomial fitting of the refractive index data from Ref. [43](Fig. 7b). The simulation results in Fig. 7c exhibit that using a BaF_2_ layer with a thickness of 0.25 µm as the anti-reflection coating layerunder the substrate., the responsivity at a bias of − 0.2 V and the radiation power of 0.05 W/cm^2^ can be enhanced by about 98.5% compared to the case without anti-reflection coating layer; the quantum efficiency also increases significantly (Fig. 7c).

## Conclusion

This work demonstrates the combination of composition grading and δ-doping as an effective band engineering method to offer an InAsSb nBn detector for high-performance MWIR applications. We used this method to reduce the valence band offset in nBn structures with InAs_0.81_Sb_0.19_ alloy absorber. Such detectors have much lower dark current than conventional detectors and significantly improved device performance can be obtained by suppressing dark current components due to SRH, BTBT, and TAT. At a temperature of 150 K, the simulation results of our proposed nBn detector have shown that the peak current responsivity at a wavelength of 4.5 μm is 1.6 A/W, and a quantum efficiency of more than 48% has been achieved. Also, the specific detectivity was about 3.37 × 10^10^ cm⋅Hz^1/2^/W at 150 K with 0.05 W/cm^2^ radiation and − 0.2 V bias voltage. To reduce the effects of infrared reflection from the lower surface of the nBn detector, we designed an anti-reflection coating layer of BaF_2_ with a thickness of 0.5 μm under the substrate, which has led to a better performance of the nBn detector in the simulation. This design significantly increased the responsivity and quantum efficiency of the proposed photodetector.

## Data Availability

The data that support the findings of this study are available from the corresponding author, upon reasonable request.
